# Relationship of Internet gaming disorder with dissociative experience in Italian university students

**DOI:** 10.1186/s12991-018-0198-y

**Published:** 2018-06-15

**Authors:** Concetta De Pasquale, Carmela Dinaro, Federica Sciacca

**Affiliations:** 1 Department of Education Science, University of Catania, Via Teatro Greco, 84, Catania, Italy; 2 Drug Addiction Health Service, SER.T-ASP3 in Catania, Via Fabio, 1, Acireale (CT), Italy

**Keywords:** Addiction, Dissociative experience, Young adults, Internet gaming disorder

## Abstract

The purpose of this study was twofold: (a) to investigate the prevalence of Internet gaming disorder (IGD) among Italian university students and (b) to explore the associations between the former and dissociative phenomena. The sample included 221 college students, 93 males and 128 females, aged between 18 and 25 (*M* = 21.56; SD = 1.42). They were asked to state their favourite games choice and were administered a demographic questionnaire, the APA symptom checklist based on the diagnostic criteria of IGD in the DSM-5, the Internet Gaming Disorder Scale Short Form (IGD9-SF) and the Italian version of dissociative experience scale for adolescents and young adults. The different game types used are distributed as follows: Massively Multiplayer Online Role-Playing Game (30%), flash games (26%), multiplayer games (24%), and online gambling (23%). The results of the study showed a high incidence of Internet gaming disorder risk in college students (84.61%). Specifically, our data confirmed the literature on the incidence of the male gender bias among online players (*M* = 28.034; SD = 2.213). Thirty-three subjects (31 male and 2 female) on 221 (14.9%) matched five or more criteria for clinical diagnosis of IGD. The data showed a positive correlation between Internet gaming disorder risk and some dissociative experiences: depersonalisation and derealisation (AbII/item6 *r* = .311; DD/item6 *r* = .322); absorption and imaginative involvement (AbII/item2 *r* = .319; AbII/item8 *r* = .403) and passive influence (PI/item3 *r* = .304; PI/item4 *r* = .366; PI/item9 *r* = .386). This study shedded light on psychopathological aspects that preceded the spread of IGD and encourages the implementation of a programmatic plan of preventative interventions by Italian public institutions, to prevent and tame the spreading of such addictive behaviours.

## Introduction

The online game in contemporary society is a problem that should not be underestimated. Selnow stated that video games are a sort of “electronic friend” that provide the company and satisfy the relational needs of an individual [1].

The game has been a human life’s constant since the past times. Callois revised the anthropological concept of game, describing it as a free and voluntary activity, source of joy and fun that can have a negative aspect; if the game goes beyond its limits, if it loses its free character and separated from reality, it can only produce “derived” and “corrupted” forms [2].

The widespread diffusion of technological devices has affected the gaming practices as well. Indeed, in recent times, digital and online gaming became a common form of entertainment, growing in popularity. This definition encompasses games requiring a connection, on multiple platforms and a variety of interactions with other players. Online gaming differs from its offline version in the central role given to a nonhuman entity setting out the rules and deciding upon players’ state in the game. According to the last report of the Entertainment Software Association (ESA), 31% of gamers are under the age of 18 and 60% are male [3].

Massively Multiplayer Online Role-Playing Game (MMORPG), multiplayer games, flash games, and online gambling (especially online poker) are the most consumed categories of games played online worldwide.

MMORPG are online role-playing games, themed mainly around fantasy narratives or conflict scenarios. They involve the creation of a digital avatar, defining its identity and appearance, and taking place in virtual worlds where players interact with each other. The multiplayer dimension is realized through the hosted connection of multiple individual machines to a server, be it consoles, personal computers or mobile devices.

Depending on the case, participation may be simultaneous, with all players in action at the same time, or asynchronous, when players’ interaction is not contemporaneous.

Flash games are played directly from the browser without the need to download additional programs.

Online gambling games are digital transpositions of traditional gambling; their novelty comes from the monetary dimension attached to games, intertwined with risk and prediction of uncertain outcome.

To form a conceptual framework, it is useful to refer to Griffith [4], who suggests considering online gaming addiction in the same light as drugs and other addictive substances. Indeed, subsequent research confirmed that engrossed players’ brain shares similar features with that of drug addicts.

Along this similarity, the technological addictions are characterized by the central role of a technological device in thoughts and affections of the individual, by its ability to swing mood tone towards positive and rewarding experiences [5, 6]. In more detail, the neurobiological bases of gaming addiction are similar to those of the chemicals’ addiction [7, 8]. In the gaming addict, as well as in the drug addict, it is possible to notice the high levels of dopamine, norepinephrine and a high presence of endorphins [9]. Holden [10] has also verified that pathological videogames players show changes of brain activity in the limbic and frontal systems similar to those occurring in cocaine addicts, namely a significant reduction of white substance in the orbitofrontal and inferior fronto occipital area. According to recent psychiatric nosography, the Internet gaming disorder (IGD) appears in the DSM-5 in Section III as a condition that is not yet classified as a formal disorder, but requires further research and clinical investigation [11].

DSM-5 proposes nine criteria to diagnose IGD: (1) Preoccupation with Internet games: the individual thinks intensively about previous gaming activity or anticipates playing the next game as Internet gaming becomes the dominant activity in daily life; (2) Withdrawal symptoms when Internet gaming is absent (typically described as irritability, anxiety, or sadness, but there are no physical signs of pharmacological withdrawal.); (3) Tolerance: the need to spend increasing amounts of time engaged in Internet games; (4) Unsuccessful attempts to control the participation in Internet games; (5) Loss of interests in previous hobbies and entertainment as a result of, and with the exception of, Internet games; (6) Continued excessive use of Internet games despite knowledge of psychosocial problems; (7) Has deceived family members, therapists, or others regarding the amount of Internet gaming; (8) Use of Internet games to escape or relieve a negative mood (i.e. feelings of helplessness, guilt, anxiety); (9) Has jeopardized or lost a significant relationship, job, or educational or career opportunity because of participation in Internet games.

A strand of the literature highlights the association between psychiatric disorders and Internet gaming disorder, showing a correlation between IGD, dissociation and mood disorders (depression, anxiety) [12–18].

Starting from these premises, the purpose of this study was twofold: (a) to investigate the prevalence of Internet gaming disorder (IGD) among Italian university students and (b) to explore the associations between the former and dissociative phenomena.

## Materials and methods

### Study sample

The sample consisted of 240 university students recruited from different departments of University of Catania (Italy) over the period from March 2017 to December 2017.

All students gave written informed consent before being included in the study.

Data were collected in two separate sessions. In the first stage, students were administered the demographic questionnaire, asked their favourite games choice, and administered the Symptom Checklist-90 Revised (SCL-90 R). Derogatis’ Symptom Checklist (SCL-90R) was applied to screen for psychopathological syndromes and to seize the students’ clinical level of distress. In the second stage, we conducted a diagnostic interview following the diagnostic criteria of IGD in the DSM-5 and students were administrated the Internet Gaming Disorder Scale Short Form (IGD9-SF) alongside with the Italian version of dissociative experience scale for adolescents and young adults (A-DES).

The Symptom Checklist-90 Revised (SCL-90 R) [19] questionnaire shows that 19 participants display clear psychopathological conditions, such as depression and psychosis. Hence, we excluded them from the sample to avoid pollution of group-level results. The final sample of the study includes 221 college students, 93 males and 128 females, aged between 18 and 25 (*M* = 21.56; DS = 1.42). The sample was drawn from the following departments: Architecture = 6%, Education Sciences = 28%, Engineering = 18%, Physics = 11%, Psychology = 32%, Agricultural Sciences = 5%.

### Measures and procedure

The protocol we employed consists of a demographic questionnaire gaming preferences, the IGD9-SF, a diagnostic interview targeting IGD as of the DSM-5 criteria, and the Italian version of dissociative experience scale for A-DES.

The IGD9-SF [20] was a short psychometric instrument that defines the nine basic criteria according to the DSM-5 [11], developed by Orsolya Kiraly and Zsolt Demetrovics, of the Institute of Psychology Eotvos Lorand University, and Halley M. Pontes and Mark D. Griffiths, of the University of Nottingham. The answers to the nine questions composed this test are structured in a Likert scale of five points: 1 “never”, 2 “rarely”, 3 “sometimes”, 4 “often”, 5 “very often”.

The IGD9-SF allowed the evaluation of the disorder severity by summing the gamers’ answers. This score can range from 9 to 45, with higher scores pointing to higher degrees of gaming disorder. In addition, the scale allowed a more granular classification: we define disordered gamers as those falling in the bin from 36 to 45 points, while non-disordered gamers belong in the remaining score range, from 9 to 35 [21]. We evaluated the instrument reliability using Cronbach’s alpha (*α* = .87).

To diagnose the presence of IGD, we administered the APA symptom checklist containing the nine IGD criteria in “yes/no” format [21]. The disorder was diagnosed when more than five symptoms out of nine are self-reported, in line with the DSM-5 [11]. We ranked the Internet gaming disorder effects according to its disruption on normal activities, from mild, to moderate, to severe. Individuals reporting severe Internet gaming disorder spend more hours on the computer and reported losses or lack of interest in social activities.

The Italian version of dissociative experience scale for A-DES consisted of 30 questions on everyday life experiences [22]. Participants are asked to state the frequency of experiencing a specific situation (from 0 = never to 10 = always). This version was an adaptation of the one developed at the University of Arkansas [23]. The A-DES is divided into four subscales: Dissociative amnesia (DA), absorption and imaginative involvement (AbII), depersonalization and derealisation (DD), and passive influence (PI). Dissociative amnesia (DA) was characterized by one or more episodes of inability to remember important personal information, usually too relevant to be explained with ordinary forgetfulness. Absorption and imaginative involvement (AbII) concerned an involvement, leading to neglecting the surrounding environment. Depersonalization–derealization (DD) disorder occurred when the subject persistently or repeatedly feels as a disembodied observer of her activities or lacks confidence in the actual reality of her environment, or any combination of the former symptoms. Passive influence (PI) has been defined as the tendency to feel dispossessed of one own feelings, thought and behaviours, as if these were forcedly imposed by an external source [11].

We reported A-DES reliability using Cronbach’s alpha (*α* = .95). In addition, the reliability of the individual subscales was the following: DA = .76, AbII = .66, DD = .85, PI = .74, while split-half correlation is DA = .63, AbII = .48, DD = .67, PI = .57 [22].

### Statistical analyses

We ran a set of statistical analyses on our data, namely *t* test on group differentials, factors correlation, and multivariate linear regression and instruments reliability was assessed with Cronbach’s alpha. We employed SPSS 24 in this study.

## Results

The descriptive analysis showed that the 73% of sample declared to be a player and use more than one type of game. The different game types used are distributed as follows: MMORPG (30%), flash games (26%), multiplayer games (24%), online gambling (23%).

The APA symptoms checklist identified that 33 out of 221 subjects reported at least 5 of the aforementioned IGD symptoms, corresponding to 14.9% of the whole sample: this subset was largely composed by men, 31 out of 33.

Our sample presented a dominant share of high scores in the IGDS9-SF test (84.61%), suggesting the presence of relevant IDG risks. The total scores in the IGD9-SF (compared with the normative cut-offs previously described in the “Measures and procedure” section) showed relevant gender differences towards gaming. In comparison with women, men showed a significantly higher risk of IGD, as shown in the last line of Table 1, which summarizes the most relevant results of the IGD risk gender differences.

**Table 1:** Comparison of male and female subsamples on internet gaming disorder (IGD9-SF test)

IGD9-SF	University students (*N* = 221)					
	Male		Female		*t*	*p*
	*M*	SD	*M*	SD		
1. Do you feel preoccupied with your gaming behaviour?	3.0860	.40796	3.0000	.00000	2.3870	.0178
2. Do you feel more irritability, anxiety or even sadness when you try to either reduce or stop your gaming activity?	3.0645	.35528	3.0156	.17678	1.3455	.1799
3. Do you feel the need to spend increasing amount of time engaged gaming in order to achieve satisfaction or pleasure?	3.1935	.59450	3.0156	.17678	3.1989	.0016
4. Do you systematically fail when trying to control or cease your gaming activity?	3.1613	.53751	3.0000	.00000	3.3980	.0008
5. Have you lost interests in previous hobbies and other entertainment activities as result of your engagement with the game?	3.0430	.29170	3.0156	.17678	.8664	.3872
6. Have you continued your gaming activity despite knowing it was causing problems between you and other people?	3.0430	.29170	3.0156	.17678	.8664	.3872
7. Have you deceived any of your family members, therapists or others because the amount of your gaming activity?	3.0217	.20851	3.0000	.00000	1.1785	.2399
8. Do you play in order to temporarily escape or relieve a negative mood?	3.3656	.77719	3.2188	.62667	1.5527	.1219
9. Have you jeopardized or lost an important relationship, job or an educational or career opportunity because of your gaming activity?	3.0860	.40796	3.0156	.17678	1.7413	.0830
Total score	28.034	2.2138	27.293	.7566	3.5124	.0005

IGD9-SF: Internet Gaming Disorder Scale Short Form; *N*: sample number; *t*: *t* test statistic; *p*: probability value; *M*: mean; SD: standard deviation

Specifically, men showed significant differences in item 1 (*p* = .0178), 3 (*p* = .001) and 4 (*p* = .000) which is related to the expression of alertness and apprehension, the difficulty of controlling gaming activity and growing need to play to achieve satisfaction and pleasure.

Concerning the results obtained in A-DES, the descriptive analysis of our sample showed a significant presence (*p* < .05) of dissociative experiences in men (*M* = 50.15; SD = 35.25) resulting from the sum of the underlying subscales: AbII (*M* = 14.5699; SD = 9.65536), DD (*M* = 14.8280; SD = 13.23338), PI (*M* = 9.6667; SD = 8.18845), as shown in Table 2.

**Table 2:** Comparison of male and female subsamples on dissociative experience (A-DES)

A-DES	University students (*N* = 221)					
	Male		Female		*t*	*p*
	*M*	SD	*M*	SD		
DA	11.0860	9.78537	8.8189	9.45534	1.7340	.0843
AbII	14.5699	9.65536	10.9375	8.36072	2.9862	.0031
DD	14.8280	13.23338	10.2756	13.37712	2.5089	.0128
PI	9.6667	8.18845	6.8898	7.11161	2.6878	.0077
Total score	50.1505	35.25927	36.9764	34.36440	2.7829	.0059

A-DES: dissociative experience scale for adolescent and young adult; *N*: sample number; DA: dissociative amnesia; AbII: absorption and imaginative involvement; DD: depersonalization and derealisation; PI: passive influence; *t*: *t* test statistic; *p*: probability value; *M*: mean; SD standard deviation

Table 3 reported the Pearson correlations between A-DES subscales scores and IGD9-SF test. Specifically, the increasing time spent playing, the loss of control, and daily activities impairment correlated with loss of awareness of the surrounding environment (AbII/item3 *r* = .446; AbII/item4 *r* = .314; AbII/item9 *r* = .341; DD/item9 *r* = .293), with a sense of foreignness from their feelings, thoughts and behaviours (PI/item3 *r* = .304; PI/item4 *r* = .366; PI/item9 *r* = .386) and with episodes of amnesia (DA/item3 *r* = .264; DA/item4 *r* = .323). Moreover, relational difficulties due to the game correlated significantly with experiences of depersonalisation and derealisation (AbII/item6 *r* = .311; DD/item6 *r* = .322), with episodes of dissociative amnesia up to the moments of extraneousness towards oneself (DA/item6 *r* = .281; PI/item6 *r* = .277). Absorption and imaginative involvement episodes correlate with states of anxiety, irritability and emotional fragility, when trying to reduce or stop playing (AbII/item2 *r* = .319; AbII/item8 *r* = .403). Furthermore, weaker but equally interesting correlations have been highlighted. Specifically, loss of interest in previous hobbies and other entertainment activities and the temporarily need to escape or relieve a negative mood correlated with episodes of dissociative amnesia for past or newly acquired events (DA/item5 *r* = .245; DA/item8 *r* = .267), with sense of foreignness from own feelings and behaviours and loss of awareness of the surrounding environment (AbII/item5 *r* = .228; PI/item5 *r* = .271; DD/item5 *r* = .267; PI/item8 *r* = .273).

**Table 3:** Correlations (Pearson *r*) between A-DES scores and IGD9-SF scores

A-DES	University students (*N* = 221)								
	IGD9-SF								
	Item 1	Item 2	Item 3	Item 4	Item 5	Item 6	Item 7	Item 8	Item 9
DA	.083	.165	.264**	.323**	.245**	.281**	.170	.267**	.299
AbII	.288**	.319**	.446**	.314**	.228**	.311**	.234**	.403**	.341**
PI	.138	.206	.304**	.366**	.271**	.277**	.151	.273**	.386**
DD	.003	.150	.164	.230**	.267**	.322**	.177	.158	.293**

*N*: sample number; A-DES: dissociative experience scale for adolescent and young adult; DA: dissociative amnesia; AbII: absorption and imaginative involvement; PI: passive influence; DD: depersonalization and derealisation; IGD9-SF: Internet Gaming Disorder Scale Short Form

** *p *< *.05*

## Discussion and conclusions

This study shedded light on the high incidence of Internet gaming disorder risk in college students assessed in our research (84.61%). Specifically, our data were in line with the literature consensus on the male gender bias among online players.

Sport, physical combat competitive spirit, and the challenge are the fundamental features of online games and are, therefore, the elective choice of male students. Men are especially prone to the charm of power and sublimated conflict [24] that these kinds of games allow: it might be considered as a symbolic way to test one’s skills and capacity. While many other reasons concur in the explanation of gaming addiction, it is clear that some applications make this addiction more likely.

The high significance displayed by items 1, 3, and 4 of the IGD9-SF test leads us to point out how online players misperceive their activity. In fact, what they consider central and crucial in their life is nothing more than a pastime: this misconception prevents them from realizing their drift away from other activities, as much as their enormous use of network traffic.

They do not cultivate hobbies, sports, and grow ever farther away from consolidated friendships in the actual world. These are replaced by those established online, gradually implementing a sort of oblivion from the surrounding world. Testing and showing off their personal skills is a twofold activity. On one hand, online players value it as a pleasant experience and, on the other hand, this might lead to a gradual and unconscious disconnection from reality, replaced by a fictive self that gains relevance in one’s personality. This latter phenomenon eventually leads to a more fragile control over the individual’s life and her self-awareness. The correlation between Internet gaming disorder risk and some dissociative experiences that emerge from our study supports this hypothesis. As shown by the correlations with items 5, 6, 8 and 9 of IGD0-SF if the immersion of the online player in the virtual world becomes predominant, it can turn into a mode of alienation, losing awareness of the surrounding environment, with disconnection from one’s feelings, thoughts and behaviours and with experiences of amnesia. Vulnerable people presenting disorders of affective and emotional regulation are obviously more exposed to the risk of Internet gaming disorder, as with other addictions. These subjects are more prone to crave for increasing levels of pleasure and involvement experienced within the gaming environment, which serves a playful and cathartic function at once. Online gaming becomes a device to handle anxiety and tension generated by the existential circumstances, to cope with isolation, social withdrawal, frustration, and life dissatisfaction [25–28].

Until recently, the literature formed a growing consensus on the interactions of comorbidity (including depression, anxiety and psychopathology in general), with Internet gaming disorder risk. These factors suggestively contribute to increase the vulnerability towards a problematic use of Internet [12].

We believe that this study provides interesting insights into aspects to be addressed as the first signs of a severe psychopathological condition [29].

Based on these results, we encourage the implementation of a programmatic prevention plan by Italian public health and education institutions to anticipate and limit the spread of online gaming addiction.

The educational, healthy, use of the Web may be taught as a school subject in itself, having a strong transdisciplinary connotation.

These interventions gain their effectiveness when they spur a search for authentic, real-life experiences enriching one’s identity through genuine interaction with other people, tight daily and weekly scheduling of activities. The latter permit to steer away from detrimental habits like disproportionate consumption of videogames and technological devices, like smartphones or tables. It is necessary to balance the activities of the online gaming with studying, reading and social activities.

It is important to support a preventive education that should involve family members, public institutions and the society as a whole to promote a culture of desire keeping contact with the real “principle of duty”.

However, a viable psycho-pedagogical process should be modelled ad hoc for groups of young adults, with the objective of developing cognitive behavioural strategies aimed at removing those risky thoughts and actions, which prevent these individuals from operating positive changes in their life [30, 31].
